# Genome-wide identification of microRNAs and their targets in wild type and *phyB* mutant provides a key link between microRNAs and the phyB-mediated light signaling pathway in rice

**DOI:** 10.3389/fpls.2015.00372

**Published:** 2015-05-29

**Authors:** Wei Sun, Xiao Hui Xu, Xiu Wu, Yong Wang, Xingbo Lu, Hongwei Sun, Xianzhi Xie

**Affiliations:** ^1^Shandong Rice Research Institute, Shandong Academy of Agricultural SciencesJinan, China; ^2^Shandong Key Laboratory of Plant Virology, Institute of Plant Protection, Shandong Academy of Agricultural SciencesJinan, China; ^3^Shandong Academy of Agricultural SciencesJinan, China

**Keywords:** rice, phytochrome B, light signaling, high-throughput sequencing, MicroRNA, degradome analysis, transcription factor

## Abstract

Phytochrome B (phyB), a member of the phytochrome family in rice, plays important roles in regulating a range of developmental processes and stress responses. However, little information about the mechanisms involved in the phyB-mediated light signaling pathway has been reported in rice. MicroRNAs (miRNAs) also perform important roles in plant development and stress responses. Thus, it is intriguing to explore the role of miRNAs in the phyB-mediated light signaling pathway in rice. In this study, comparative high-throughput sequencing and degradome analysis were used to identify candidate miRNAs and their targets that participate in the phyB-mediated light signaling pathway. A total of 720 known miRNAs, 704 novel miRNAs and 1957 target genes were identified from the fourth leaves of wild-type (WT) and *phyB* mutant rice at the five-leaf stage. Among them, 135 miRNAs showed differential expression, suggesting that the expression of these miRNAs is directly or indirectly under the control of phyB. In addition, 32 out of the 135 differentially expressed miRNAs were found to slice 70 genes in the rice genome. Analysis of these target genes showed that members of various transcription factor families constituted the largest proportion, indicating miRNAs are probably involved in the phyB-mediated light signaling pathway mainly by regulating the expression of transcription factors. Our results provide new clues for functional characterization of miRNAs in the phyB-mediated light signaling pathway, which should be helpful in comprehensively uncovering the molecular mechanisms of phytochrome-mediated photomorphogenesis and stress responses in plants.

## Introduction

Light is not only a source of energy for photosynthesis, but also a key environmental factor that affects plant growth and development. To adapt to changing environments, plants perceive light signals through an array of photoreceptors to make appropriate adjustments (Chen et al., 2004; Franklin, 2009). Phytochrome (phy) is a kind of photoreceptor that mainly perceives and responds to the red and far-red light regions. *In vivo*, native phytochromes form homodimers and heterodimers, and exist in two reversible conformations under different light spectrums (Rockwell et al., 2006). In etiolated seedlings, phytochromes are present as the red-light-absorbing form (Pr, biologically inactive) in the cytoplasm. However, red light induces a conformation shift from Pr to the far-red-light-absorbing form (Pfr, biologically active) and promotes translocation of the Pfr form into the nucleus (Yamaguchi et al., 1999; Chen et al., 2005). In the nucleus, phytochromes interact with other nuclear proteins and trigger transcription cascades to regulate light responses.

Rice, one of the most important cereals in the world, has been used as a monocot model plant for molecular studies. Three genes (*PHYA*, *PHYB*, and *PHYC*) encoding phytochromes have been identified in the rice genome (Kay et al., 1989; Dehesh et al., 1991; Basu et al., 2000; Xie et al., 2014). In etiolated rice seedlings, phyA is the most abundant phytochrome, whereas phyB is the predominant phytochrome under white light (Xie et al., 2014). Previous studies have revealed that phyB-mediated light signaling regulates multiple aspects of plant growth and development, such as seedling establishment, plant architecture, and flowering time (Izawa et al., 2002; Takano et al., 2005; Zhao et al., 2013). Continuous red light inhibited coleoptile elongation in wild-type (WT) rice, but the inhibitory effect was significantly reduced in the *phyB* mutant, indicating that phyB plays a crucial role in photoinhibition of coleoptile elongation in rice seedlings (Takano et al., 2005). In addition, phyB-mediated red signal positively regulate chlorophyll biosynthesis and chloroplast development in rice through affecting the expression of various downstream genes (Izawa et al., 2002; Takano et al., 2005; Zhao et al., 2013). Other characteristics of rice architecture and development, such as the angle between the leaf blade and leaf sheath, root elongation and gravitropic responses, stomata development, and flowering time, were also under the control of phyB (Izawa et al., 2000, 2002; Correll and Kiss, 2005; Takano et al., 2005; Gu et al., 2011; Liu et al., 2012).

In addition to rice growth and development, phyB also participates in stress and hormone responses in rice. It was reported that the *phyB* mutant had improved drought tolerance, which was attributed to its reduced leaf area and stomata density (Liu et al., 2012). Expression analyses suggested that genes related to SA- and/or JA-dependent defense pathways were down-regulated significantly in the *phyAphyBphyC* triple and *phyAphyB* double mutants, which led to a reduction in pathogenesis-related class 1 (PR1) proteins (Xie et al., 2011, 2014). In addition, phyB also plays important roles in hormone responses. In rice, the *phyB* mutant had increased abscisic acid (ABA) content and exhibited hypersensitivity to ABA (Gu et al., 2011). Rice phyA and phyB affect the expression of the gibberellin (GA) biosynthesis gene *OsGA3ox2* and the ethylene biosynthesis gene *OsACO1* (Iwamoto et al., 2011).

MicroRNA (miRNA) is the major type of endogenous non-coding RNA in plants, and mainly functions in post-transcriptional gene silencing via mRNA cleavage or translational repression (Reinhart et al., 2002; Bartel, 2004; Taylor et al., 2014). A growing body of evidence suggests that miRNAs play key roles in plant growth and development, and stress and hormone responses by targeting specific genes (Jones-Rhoades et al., 2006; Mallory and Vaucheret, 2006; Shukla et al., 2008; Voinnet, 2009; Sunkar et al., 2012). Cup-Shaped Cotyledon1 (CUC1) and CUC2, two targets of miR164, play crucial roles in regulating meristem development and aerial organs initiation in *Arabidopsis* (Laufs et al., 2004; Mallory et al., 2004). miR156 and miR172 function antagonistically in regulating developmental transitions in both monocots and dicots (Chuck et al., 2007; Wang et al., 2009, 2011; Wu et al., 2009; Huijser and Schmid, 2011). Four miRNAs (miR159, miR165/166, miR319) participate in regulation of various developmental processes by targeting the GAMYB, Homeodomain Leucine Zipper (HD-ZIP) and TCP transcription factors in *Arabidopsis*, respectively (Millar and Waterhouse, 2005; Jones-Rhoades et al., 2006; Mallory and Vaucheret, 2006; Jung et al., 2009; Rubio-Somoza and Weigel, 2011). A number of well-conserved plant miRNAs are directly involved in auxin signaling, such as miR160, miR164, miR167, miR390, and miR393 (Mallory et al., 2004; Guo et al., 2005; Wang et al., 2005; Yang et al., 2006; Marin et al., 2010; Si-Ammour et al., 2011). Recent studies revealed that environmental stresses, including both biotic and abiotic stresses, induce differential expression of a number of miRNAs. For example, abiotic stresses, drought and salinity, could induce the differential expression of a variety of miRNAs in different plant species, such as miR156, miR160, miR397, and miR402 (Sunkar and Zhu, 2004; Ding et al., 2009; Zhou et al., 2010; Kantar et al., 2011). A number of miRNAs are induced when plants suffer from pathogenic fungi, bacteria, and viruses infection (Ruiz-Ferrer and Voinnet, 2009; Katiyar-Agarwal and Jin, 2010).

As far as we know, little information about the phyB-mediated light signaling pathway has been reported in rice, although phyB plays important roles in rice developmental processes and stress responses. Moreover, only one miRNA acting downstream of phyB was identified in potato (*Solanum tuberosum* L.) (Martin et al., 2009), and no more information about miRNA roles in the phyB-mediated light signaling pathway has been reported in other flowering plants. To identify miRNAs and their targets involved in the phyB-mediated light signaling in rice, we performed high-throughput miRNA sequencing and degradome analysis on WT and *phyB* mutant rice. Analysis of the targets of differentially expressed miRNAs revealed that miRNAs probably participate in phyB-mediated light signaling mainly via down-regulating various transcription factor families.

## Materials and methods

### Plant materials and growth conditions

WT rice and the *phyB* mutant used in this study are *Oryza sativa* L. cv. Nipponbare. The *phyB* mutant is *phyB1* allele as described by Takano et al. (2005). The *phyB1* mutant was isolated from γ-ray-mutagenized M2 plants by phenotype screening. The mutant has an insertion of one nucleotide at the position of 481st amino acid to cause an early stop.

Seeds of WT rice and the *phyB* mutant were surface-sterilized and cultured as previously described (Liu et al., 2012). The plants were grown in a greenhouse with natural light conditions and controlled temperature (28°C, day/25°C, night) during July in Jinan, Shandong, China (latitude 36°40′N; longitude 117°00′E). At the five-leaf stage, the *phyB* mutant exhibited obvious different phenotypes from WT, such as larger declination angles of leaves, shorter plant height, wider leaf blade, reduced stomata density and transpiration rate (Takano et al., 2005; Liu et al., 2012). In addition, leaf is also an organ to measure day length and generate a mobile flowering signal to regulate photoperiodic flowering in rice (Tsuji et al., 2011). Therefore, the leaf is an important organ for phyB-mediated photomorphogenesis and stress responses. To dissect the function of miRNAs in the phyB-mediated light signaling pathway, the fourth leaves from WT and *phyB* mutant were collected and immediately frozen in liquid nitrogen. To minimize biological variance, each sample was a mixture of leaves from four individual plants.

### Small RNA library construction, sequencing and sequencing data analysis

Total RNA was extracted using Trizol reagent (Invitrogen, Carlsbad, CA, USA) according to the manufacturer's instructions. The quantity and purity of the total RNA were determined with a Bioanalyzer 2100 and RNA 6000 Nano LabChip Kit (Agilent, Palo Alto, CA, USA) with RIN number >7.0. Small RNA libraries were generated using Illumina Trueseq Small RNA Sample Preparation kits (Illumina, San Diego, CA, USA) according to the manufacturer's guide. The purified cDNA libraries were used for cluster generation on Illumina's Cluster Station and then sequenced on HiSeq 2500 sequence analyzer at LC Sciences (Hangzhou, China). Raw reads were obtained by using Illumina's Sequencing Control Studio software version 2.8 (SCS v2.8) following real-time sequencing image analysis and base-calling by Illumina's Real-Time Analysis version 1.8.70 (RTA v1.8.70, Illumina). The sequencing data were analyzed by the ACGT101-miR pipeline script version 4.2 (LC Sciences, Houston, TX, USA).

Raw reads without inserts (adaptor sequences only) and low quality reads (reads containing more than 80% single nucleotide A/G/C/T, reads with more than two undetermined bases, reads contain stretches of A7, C8, G6, or T7), were removed from further analyses. Based on the instruction from LC Sciences, raw reads containing stretches of A7, C8, G6, or T7 were deleted because there is no reported miRNAs in plants contain stretches of A7, C8, G6, or T7. Different numbers of different bases were determined based on the statistical results of the known plant miRNAs in miRbase. The clean reads were then mapped to the rice genome to separate the unique reads. Unique reads were then used as queries to blast against the non-coding RNA sequences database (Rfam 10.1, ftp://ftp.sanger.ac.uk/pub/databases/Rfam/10.1/), and repeat-Repbase database (http://www.girinst.org/repbase/update/index.html), to identify rRNA, tRNA, snRNA, snoRNA, and repeat sequences. Then, the remaining unique reads with length ≥17 nt and ≤25 nt were used as queries to blast against miRBase version 20.0 (http://www.mirbase.org/) to identify known and novel miRNAs. Length variation at both the 3′ and 5′ ends and one mismatch inside the sequence were allowed in the alignments, but the total length of a miRNA must be ≥ 17 nt and ≤ 25 nt. If a unique read could be mapped to rice pre-miRNAs in miRBase, it was considered as known miRNAs. The remaining sequences that could not be mapped to rice pre-miRNAs in miRBase were used for novel miRNA prediction. Their flanking genome sequences were folded using RNAfold software (http://rna.tbi.univie.ac.at/cgi-bin/RNAfold.cgi) to predict secondary structures. The criteria for secondary structure prediction were: (1) number of nucleotides in one bulge in stem (≤12); (2) number of base pairs in the stem region of the predicted hairpin (≥16); (3) cutoff of free energy (kCal/mol ≤15); (4) length of hairpin (up and down stems + terminal loop ≥50); (5) length of hairpin loop (≤200); (6) number of nucleotides in one bulge in mature region (≤4); (7) number of biased errors in one bulge in mature region (≤2); (8) number of biased bulges in mature region (≤2); (9) number of errors in mature region (≤4); (10) number of base pairs in the mature region of the predicted hairpin (≥12); (11) percent of mature in stem (≥80) (Ambady et al., 2012). If a read met for all the criteria, and its normalized expression value ≥10 in at least one library, it was regarded as a novel miRNA. To determine their conservation, the novel miRNAs were used as queries to blast against the other plant species' pre-miRNAs in miRBase, and the alignment parameters were the same as that have been described above. Based on the homologies of novel miRNAs to the known miRNAs in other plant species, the novel miRNAs were classified into two groups. Group 1 novel miRNAs were orthologs of miRNAs identified in other plant species, while group 2 novel miRNAs were rice-specific miRNAs. In this study, group 2 miRNAs were named as 5p- or 3p- miRNAs, depending on their location, at the 5′-end or 3′-end of the hairpin structures. However, novel miRNAs in group 1 were named as 5p/3p or p5/p3 miRNAs. Both the 5p/3p and p5/p3 novel miRNAs in group 1 could not be mapped to rice pre-miRNAs in miRbase, but could be mapped to the precursors of miRNAs in other plant species. If they show high similarities to miRNAs in other plants, they were named as 5p/3p novel miRNAs, if they show high similarities to the potential plant (except rice) miRNAs that located on the other arms of hairpin structures with known plant miRNAs, they were named as p5/p3 novel miRNAs.

Differentially expressed miRNAs between the WT and *phyB* were identified by IDEG6 (http://telethon.bio.unipd.it/bioinfo/IDEG6_form) using Fisher's exact test and chi-squared 2 × 2 methods based on normalized deep-sequencing counts. The sequencing data were submitted to the NCBI GEO database (http://www.ncbi.nlm.nih.gov/geo) under the accession number GSE62334.

### Degradome library construction, sequencing, and analysis

Two degradome libraries were constructed using the fourth leaves of the WT and *phyB* mutant at five-leaf stage following methods described recently (Ma et al., 2010), and sequenced on HiSeq 2500 sequence analyzer at LC Sciences (Hangzhou, China). The CleaveLand 3.0 software package (Addo-Quaye et al., 2009a,b) and the ACGT101-DEG program (LC Sciences, Houston, TX, USA) were used to identify candidate targets of the known and novel miRNAs. miRNAs identified in this study were aligned to unique reads resulted from degradome sequencing, and these alignments were then scored according to a previously described scheme developed for plant miRNA/target pairings (Allen et al., 2005). If the alignment score ≤4, and at least one raw read was detected at the cleavage site, it was considered as a candidate target transcript. All targets were grouped into five categories based on the abundance of the resulting mRNA tag relative to the overall profile of degradome reads that matched the target (Yang et al., 2013). Category “0” comprised transcripts with >1 raw read at the position, with abundance at a position equal to the maximum on the transcript, and with only one maximum on the transcript. Category “1” is defined as >1 raw read at the position, with abundance at the position equal to the maximum on the transcript, and more than one maximum position on the transcript. Category “2” is defined as >1 raw read at the position, and abundance at the position less than the maximum but higher than the median for the transcript. Category “3” contained transcripts with >1 raw read at the position, and abundance at the position equal to or less than the median for the transcript, and transcripts in category “4” showed only one raw read at the position. Gene Ontology (GO) analysis of the candidate target genes of known and novel miRNAs was carried out using the AgriGO toolkit (http://bioinfo.cau.edu.cn/agriGO/).

### Real-time reverse transcription PCR (qRT-PCR)

Total RNAs were extracted from the fourth leaves of the WT and *phyB* plants as described above. Genomic DNA contamination was eliminated by RNase-free DNase I treatment (Promega, Madison, WI, USA). For miRNA qRT-PCR, miRNA first-strand cDNA was synthesized using the miRcute miRNA First-Strand cDNA Synthesis kit (Tiangen, Beijing, China). The expression levels of 15 random selected miRNAs were quantified by qRT-PCR with the SYBR PrimeScript™ miRNA RT-PCR kit (Tiangen, Beijing, China). For targets qRT-PCR, first-strand cDNA was synthesized using M-MLV reverse transcriptase according to the manufacturer's instructions (Promega, Madison, WI, USA). The expression patterns of selected genes were detected using SYBR Green Real-time PCR Master Mix (PE Applied Biosystems, Foster City, CA, USA) on the Stepone Plus system (Applied Biosystems, USA). The primers used in qRT-PCR are listed in Additional file 1. Three biological replicates were included for each miRNA and target gene in qRT-PCR analysis. The relative expression ratios of each miRNA and target gene were calculated using the delta-delta threshold cycle relative quantification method with the internal control of 5.8s rRNA and *OsEF-1α*, respectively.

## Results

### Small RNA profiles of WT rice and the *phyB* mutant

To identify miRNAs involved in phyB-mediated light signaling, two independent small RNA libraries constructed with RNAs from the WT and *phyB* mutant leaves were sequenced. A total of 13,334,846 and 10,941,196 raw reads were generated from the WT and *phyB* mutant libraries, respectively (Table 1). Adaptor sequences and low quality reads were then removed and the remaining reads were aligned to the rice genome database, Rfam and Repeats database to filter out rRNA, tRNA, snoRNA, snRNA, other non-coding RNAs, and repeat sequences. Then, reads could be mapped to rice genome and with length ≥17 nt and ≤ 25 nt were used for further analyses. In total, there were 6,526,459 and 4,879,984 clean reads in the WT and *phyB* libraries, which corresponded to 1,426,398 and 1,247,304 unique reads respectively, were used for miRNA identification (Table 1).

**Table 1:** **Distribution of reads in the WT and *phyB* mutant small RNA libraries**.

**Libraries**	**WT**				***phyB* mutant**			
	**Total**	**Percentage of total**	**Unique**	**Percentage of unique**	**Total**	**Percentage of total**	**Unique**	**Percentage of unique**
Raw reads	13,334,846	100.00	2,610,417	100	10,941,196	100.00	2,590,954	100.00
3ADT&length filter	4,042,952	30.32	840,931	32.21	3,937,015	35.98	990,677	38.24
Junk reads	24,596	0.18	16,164	0.62	19,064	0.17	14,852	0.57
Reads have hits in Rfam database	1,641,480	12.31	92,158	3.53	1,274,246	11.65	91,631	3.54
mRNA	1,181,063	8.86	241,126	9.24	893,614	8.17	254,119	9.81
Repeats	27,930	0.21	4797	0.18	37,016	0.34	5272	0.20
rRNA	1,048,254	7.86	51,015	0.38	807,246	7.38	50,314	0.46
tRNA	432,784	3.25	22,119	0.17	367,231	3.36	22,898	0.21
snoRNA	29,425	0.22	5643	0.04	15,741	0.14	4877	0.04
snRNA	23,838	0.18	4290	0.03	8476	0.08	3412	0.03
Other Rfam RNA	107,179	0.80	9091	0.07	75,552	0.69	10,130	0.09
Clean reads	6,526,459	48.94	1,426,398	54.64	4,879,984	44.60	1,247,304	48.14

3ADT&length filter: reads removed because 3ADT was not found and lengths were <17 nt or >25 nt.

Junk reads: Junk: ≥2N, ≥7A, ≥8C, ≥6G, ≥7T, ≥10 dimer, ≥6 trimer, or ≥5 tetramer.

Rfam: a database of common non-coding RNA families except microRNA.

Repeats: Prototypic sequences representing repetitive DNA from different eukaryotic species.

There was overlap in the mapping of reads with rRNA, tRNA, snRNA, snoRNA and repeats.

**Figure 1:**
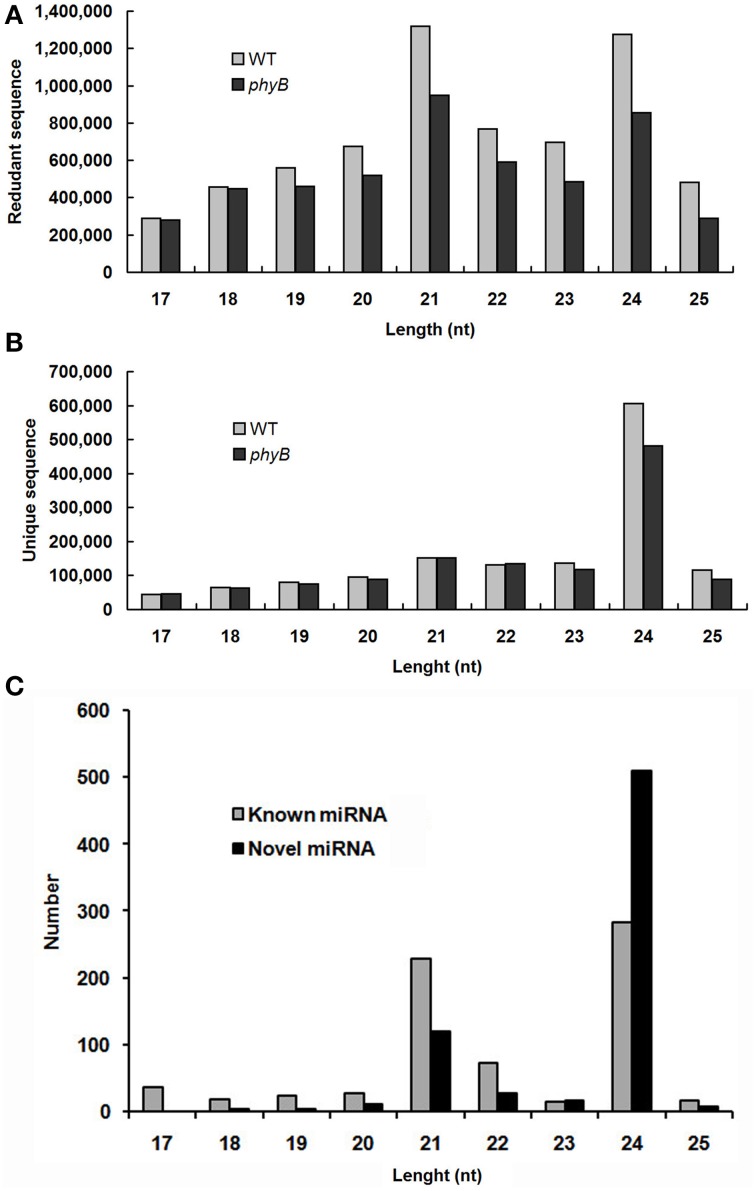
**Size distribution of small RNAs in the WT and *phyB* mutant libraries. (A)** Size distribution of redundant sequences; **(B)** Size distribution of unique sequences; **(C)** Size distribution of known and novel miRNAs identified in this study.

All small RNAs (sRNA) in the WT and *phyB* libraries showed similar size distribution patterns. The length distribution of both total clean reads and unique reads in the two libraries showed that the majority of sRNAs were 21–24 nt long, which corresponded to the typical size range of Dicer-derived products (Figures 1A,B). Size distribution analysis of the total clean reads revealed that 21 nt sRNAs were the most abundant sequences followed by 24 nt sRNAs in both the WT and *phyB* libraries (Figure 1A). However, when redundant sequences were excluded, the size distribution patterns of the sRNAs in the two libraries changed significantly. The 24 nt sRNAs represented the largest number of unique reads generated from the WT and *phyB* libraries, while 21 nt sRNAs were much less abundant (Figure 1B). It was reported that most sRNAs functioning as small interfering RNAs (siRNAs) are 24 nt long (Rajagopalan et al., 2006), the appearance of so many 24 nt sRNAs in both libraries indicated that siRNAs accounted for a large proportion in the sRNA datasets. This phenomenon has also been observed in *Arabidopsis*, rice, *Citrus trifoliata*, *Brassica campestris*, *Solanum lycopersicum*, and *Medicago truncatula* (Rajagopalan et al., 2006; Morin et al., 2008; Moxon et al., 2008; Szittya et al., 2008; Song et al., 2010; Jiang et al., 2014).

### Identification of known miRNAs in the WT and *phyB* mutant

To identify known miRNAs in the two libraries, all unique sRNA reads with length ≥17 nt and ≤25 nt were compared with rice pre-miRNAs in the miRBase v20.0 database, allowing length changes in the 5′/3′ end of the miRNA and no more than 1 mismatch in the internal sequences. Unique sequences mapping to rice miRNA precursors in miRbase v20.0 were identified as known miRNAs. Known miRNAs include two kinds of miRNAs, one is documented miRNAs in miRBase, the other one is 5p-/3p-derived miRNAs. In this study, if a miRNA that has not previously reported is located on the other arm of one hairpin structure with a documented miRNA, it was regarded as a 5p-/3p-derived miRNA. To distinguish from the documented miRNAs in miRbase, the latter one was named as -p5/-p3 miRNA in this study, as described previously (Ambady et al., 2012; Fang et al., 2013; Yang et al., 2013; Jiang et al., 2014).

In total, 720 miRNAs originating from 473 *MIRNA*s, including 251 documented rice miRNAs and 469 p5/p3 miRNAs, were identified from WT and *phyB* mutant libraries (Additional file 2a). Approximately half of these documented miRNAs (114/251) identified in this study have changes in the 5′/3′ end, or have one mismatches in the internal sequences (Additional file 2a). Different from documented miRNAs detected in this study, most of the p5/p3 miRNAs (283/469) expressed at low levels (the normalized expression value ≤10 in both libraries) (Additional file 2a).

### Identification of novel miRNAs from the WT and *phyB* mutant

It has been suggested that the formation of a stable hairpin structure is an essential prerequisite to differentiate candidate miRNAs from other sRNAs. Unique reads without matching mature rice miRNAs and/or pre-miRNAs in miRbase and with a normalized expression value ≥10 in either library were used for novel miRNA prediction.

**Figure 2:**
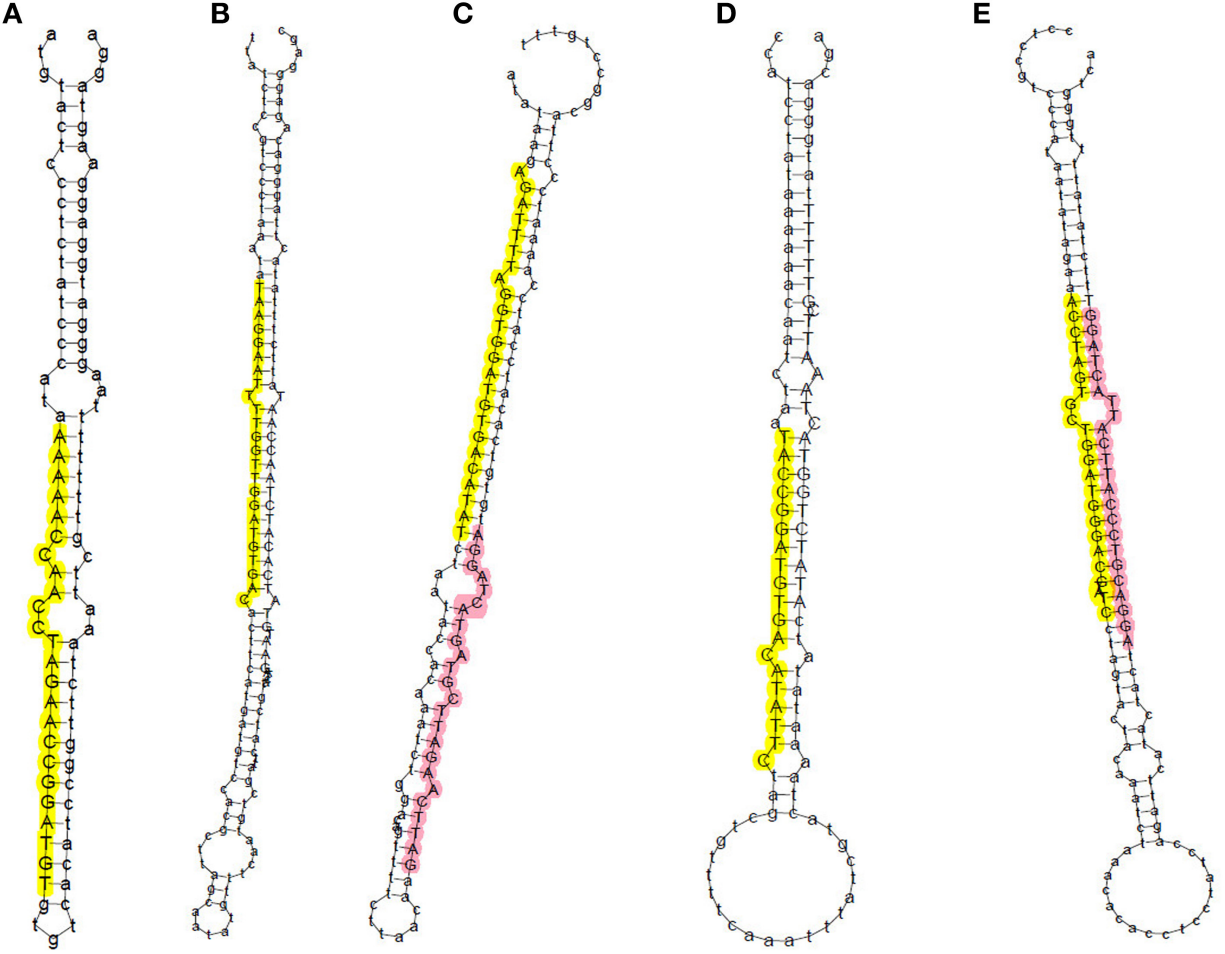
**Prediction of secondary structures of five representative novel miRNAs in rice**. Sequences in yellow and pink represent miRNAs generated from the 5′ and 3′ arm of miRNA precursors, respectively. **(A)** PC-5P-180057_8; **(B)** PC-5p-69727_33; **(C)** PC-5p-224167_6 (yellow) and PC-3p-222128_6 (pink); **(D)** PC-5p-110102_17; **(E)** PC-5p-152796_10 (yellow) and PC-3p-112150_16 (pink).

In total, 761 pre-miRNAs corresponding to 704 miRNAs were newly identified in this study (Additional file 2b). Among them, 41 novel miRNAs were orthologs of miRNAs identified in other plant species and 663 novel miRNAs were rice-specific (Additional file 2b). Most of these novel miRNAs could be detected in both WT and *phyB* library except for three novel miRNAs (PC-5p-836050_2, PC-3p-172030_8, PC-3p-123229_14), which were absent in the *phyB* library (Additional file 2b). The length distribution of these novel miRNAs varied from 17 to 25 nt, and 24 nt miRNAs (72.4%) comprised the largest category, followed by 21 nt miRNAs (16.9%) (Figure 1C, Additional file 2b). The secondary hairpin structures of five representative miRNAs were shown in Figure 2. Compared with known miRNAs, most of the novel miRNAs were expressed at relative low levels in the two libraries; 450 novel miRNAs (63.9%) had less than 20 reads in both libraries, while only 34 members (4.8%) had more than 100 reads in either of the two libraries. It was reported that new species-specific miRNAs have evolved recently, and they are usually expressed at lower levels than conserved miRNAs (Rajagopalan et al., 2006; Fahlgren et al., 2007; Yao et al., 2007). Our results were in agreement with previous findings.

Furthermore, first nucleotide bias analysis of the known and novel miRNAs showed obviously different results (Additional file 3). The first nucleotide of most known miRNAs tends to be uridine (U, 35.70%) and adenosine (A, 36.94%), whereas the novel miRNAs showed a bias for A at the 5′ terminal (60.09%) (Additional file 3). AGO proteins, the main components of the RNA-induced silencing complex (RISC), could bind to small RNAs to slice target genes (Reinhart et al., 2002; Bartel, 2004). It was reported that AGO1 is mainly involved in miRNA-mediated gene silencing pathway and preferentially recruits miRNAs with 5′ terminal U (Mi et al., 2008). However, some of miRNAs with 5′ terminal A, such as miR172, were also loaded into AGO1 (Mi et al., 2008). Thus, these miRNAs without a terminal U may be bound by AGO1 or another AGO protein with a preference for a different terminal nucleotide, as has been found in *Arabidopsis* (Mi et al., 2008).

### Identification of candidate miRNAs involved in phyB-mediated light signaling

To identify miRNAs involved in phyB-mediated light signaling, the differentially expressed miRNAs between the WT and *phyB* libraries were picked out by IDEG6 using fisher's exact test and chi-squared 2 × 2 methods. To reduce false-positive results, miRNAs with extremely low abundance (less than 10 raw reads in both libraries) were excluded from further study. For the differential expression analysis, fold change ≥2 and *q*-value ≤0.01 were used as cut-offs. As a result, a total of 135 differentially expressed miRNAs were identified, including 97 up-regulated and 38 down-regulated miRNAs in the *phyB* mutant (Additional file 4).

Among the up-regulated miRNAs, osa-miR530-5p_R+1 (a miRNA that has one base more than documented osa-miR530 in the 3′-end) was the most abundant miRNA in both libraries, and was up-regulated significantly in the *phyB* mutant (Additional file 4). Interestingly, another miRNA that derived from the 3′ arm of pre-*MIR530*, osa-miR530-3p, was also up-regulated significantly in the *phyB* mutant relative to the WT (Additional file 4). However, we have no idea whether both osa-miR530-5p_R+1 and osa-miR530-3p are functional miRNAs in phyB-mediated signaling pathway. Five members of the osa-miR169 family (osa-miR169h, osa-miR169i-3p, osa-miR169k-p3, osa-miR169r-3p, osa-miR169r-5p_R+1) were also up-regulated in the *phyB* mutant, whereas osa-miR169a-p3 was down-regulated significantly in the *phyB* mutant (Additional file 4), which suggests they may have distinct and common functions in phyB-mediated processes. Additionally, 51 novel miRNAs were up-regulated significantly in the *phyB* mutant (Additional file 4). The down-regulated miRNAs consisted of 21 known miRNAs and 17 novel miRNAs (Additional file 4). Compared with the WT, the novel miRNA PC-5p-99377_20 showed the highest down-regulation fold change in the *phyB* mutant. Five gma-miR4995 orthologous and new 5p strand miRNAs were down-regulated significantly in the *phyB* mutant (Additional file 4).

### Validation of miRNA expression by qRT-PCR

**Figure 3:**
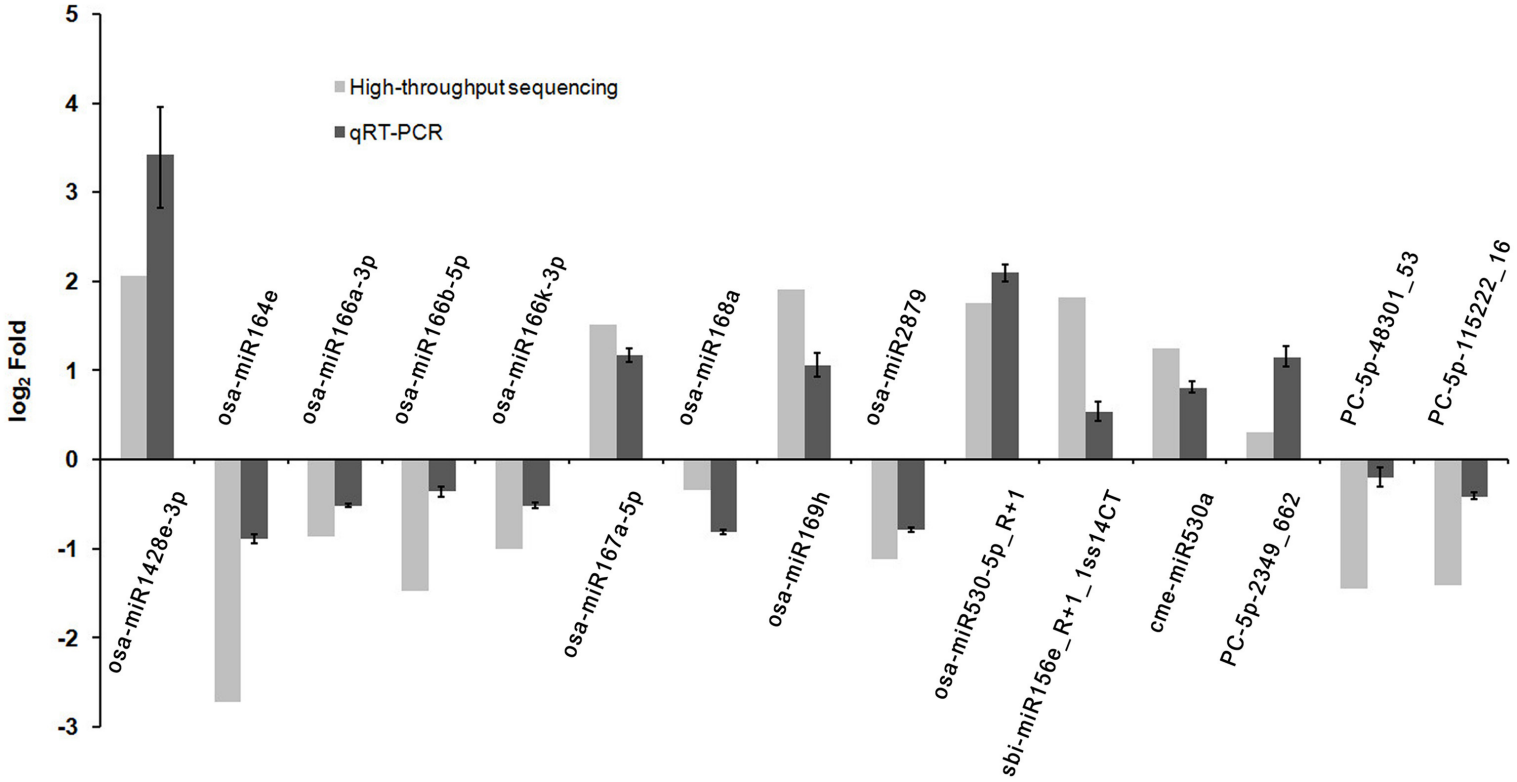
**Validation the expression of miRNAs identified from high-throughput sequencing by qRT-PCR**. Total RNAs were isolated from the fourth leaves of the WT and *phyB* mutant at the five-leaf stage. Expression of miRNAs was determined by qRT-PCR. 5.8s rRNA was used as a normalization control. Bars indicate the SD across three PCR reactions. For both high-throughput sequencing and qRT-PCR, the expression value of each miRNA in WT was set to 1, and its expression value in *phyB* mutant was calculated based on this criterion. The ratio of *phyB*/WT was log2 transformed to draw the histogram.

To verify the differential expression patterns of the miRNAs found in this study, 15 miRNAs including three novel and 12 known miRNAs were randomly selected for qRT-PCR analysis. Although most of the novel miRNAs were found to be expressed at relative low levels in the high-throughput sequencing, expression of all the three novel miRNAs was detected by qRT-PCR, highlighting the wide detection range of high-throughput sequencing. As shown in Figure 3, the expression patterns of most selected miRNAs detected by qRT-PCR were consistent with the sequencing data, such as osa-miR167a-5p, osa-miR169h, osa-miR2879, osa-miR530-5p_R+1, osa-miR1428e-3p. However, a discrepancy was also observed between the two detection methods; the expression of PC-5p-48301_53 was slightly down-regulated in the *phyB* mutant in qRT-PCR analysis whereas it was significantly down-regulated according to the sequencing data (Figure 3). This discrepancy probably caused by two reasons. Firstly, the sensitivity and specificity of the two methods are different. For a certain miRNA, qRT-PCR is probably more accurate. Secondly, we had no replicates to ensure the accuracy of the sequencing results. Overall, the results indicated that high-throughput sequencing was a powerful tool for discovering novel and differentially expressed miRNAs.

### Identification of targets of miRNAs by degradome sequencing analysis in rice

With the development of high-throughput sequencing, several methods have been established to monitor RNAs that are in the process of being degraded. Degradome sequencing is one of these high-throughput sequencing-based methods, which can identify multiple miRNA cleavage targets genome-wide in a short time (Ma et al., 2010). Therefore, we performed degradome sequencing to identify the potential cleaved mRNAs in the WT and *phyB* mutant. Compared with the WT library, more raw reads were obtained from the *phyB* mutant library (8,853,077 raw reads in WT library; 15,733,668 raw reads in *phyB* mutant library) (Additional file 5). Reads less than 15 nt after removing the 3′ adaptor were then eliminated, and the remaining raw reads were aligned to the rice genome database. As a result, 8,808,065 and 15,647,788 mappable reads corresponding to 35,328 and 36,072 transcripts were obtained from the WT and *phyB* libraries, respectively (Additional file 5).

**Figure 4:**
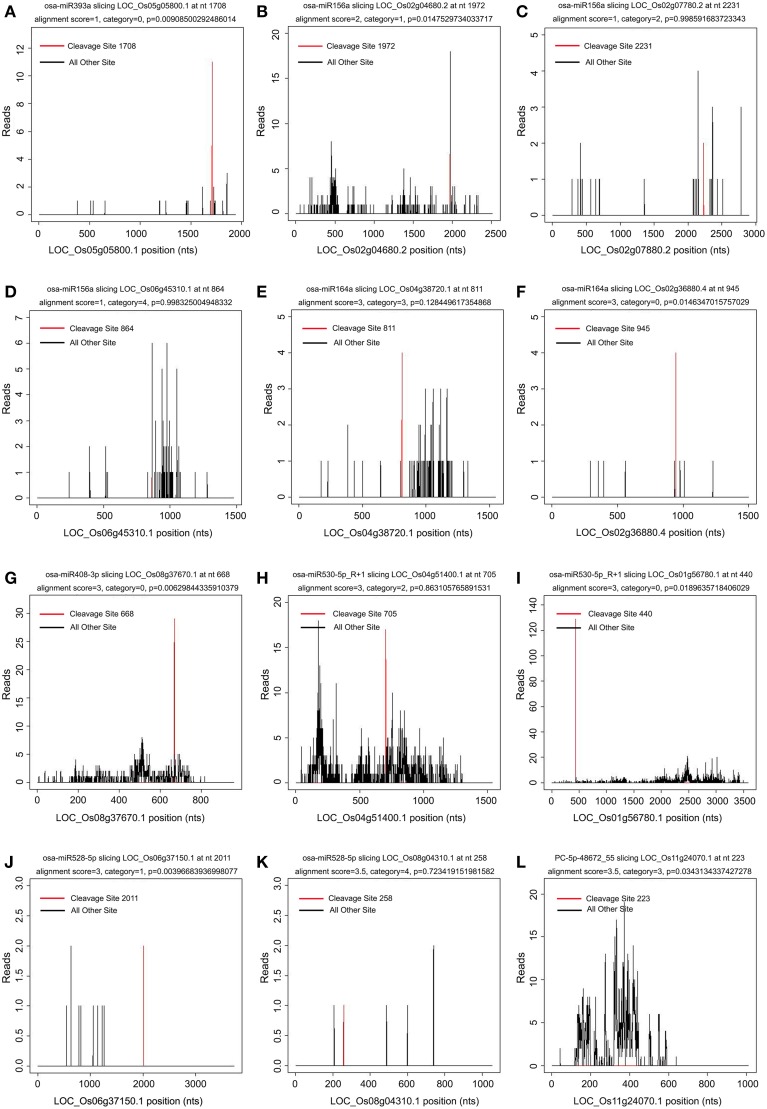
**Target plot (t-plot) of known and novel targets in different categories confirmed by degradome sequencing**. The distribution and abundance of the degradome tags along the full length of the target transcripts were shown. Black lines correspond to cleavage products, and the red line corresponds to the cleavage site predicted to be a microRNA recognition site. The categories were based on the relative abundance of the tags at the target sites. **(A–F)** t-plot of known targets cleaved by known miRNAs in the five different categories (0, 1, 2, 3, 4). **(G–L)** t-plot of novel targets in the five categories.

It was reported that the 5′ terminal nucleotides of miRNA-cleaved mRNA fragments should correspond to the tenth nucleotide of miRNA complementary sites (Llave et al., 2002; Kasschau et al., 2003). Thus, mapping degradome reads by their 5′-ends should produce a distinct peak at the cleavage site of targeted transcript. The targets of the known and novel miRNAs were identified by the CleaveLand pipeline (Addo-Quaye et al., 2009a,b) and ACGT101-DEG program (LC Sciences, Houston, TX, USA). The transcript abundance was plotted for each transcript and the sliced targets were grouped into five categories according to the relative abundance of tags at the target sites as previously described (Figure 4) (Yang et al., 2013). Both known and novel targets were observed in five categories based on t-plot analysis (Figure 4). A total of 1957 genes (2893 transcripts) were identified as targets of 254 known and 86 novel miRNAs in the two libraries (Additional file 6). Of these identified targets, 700 genes (1013 transcripts) were found in both libraries, 510 genes (776 transcripts) were found only in the WT library, and 747 genes (1104 transcripts) were found only in the *phyB* library (Additional file 6). GO analyses were then used to identify enriched GO terms among these predicted targets (Additional file 7). In the dataset of targets found in both libraries, photosynthesis was the most over-represented GO term (Additional file 7a), indicating miRNAs might regulate photosynthetic processes by slicing their corresponding targets. In addition, genes involved in stress responses and translation regulation were also over-represented in the dataset of targets found in both libraries (Additional file 7a). Similarly, these two GO terms were also enriched in the dataset of targets found only in the *phyB* library (Additional file 7b). However, few GO terms were enriched in the dataset of targets found only in the WT library (Additional file 7c).

In general, most miRNAs can cleave two or more target genes. Consistent with this, a large number of miRNAs (216/340, 63.5%) identified in this study were predicted to have more than one target (Additional file 6). For example, osa-miR156a was predicted to slice eight genes, including six genes encoding squamosa promoter-binding protein-like (SBP domain) transcription factor family proteins, one gene encoding O-methyltransferase, and one gene with unknown function. In addition, miRNAs in one family were found to slice the same targets. A gene encoding a NAC domain protein (LOC_Os02g36880) was predicted to be sliced by the osa-miR164 family (osa-miR164a/c/d/e). Interestingly, a gene encoding a nuclear transcription factor Y subunit (LOC_Os12g42400) was found to be sliced by not only the osa-miR169 family (osa-miR169b/f/h/n/r), but also by osa-MIR1430-p5, which originates from the same loci as osa-miR1430 (Additional file 6).

The targets of the novel rice-specific miRNAs were also identified by degradome sequencing analysis (Table 2). Fourteen target genes (15 transcripts) of 12 novel rice-specific miRNAs were identified in the WT library. However, the reliability of the predicted target genes was low; 11 of them fell into category 4, the rest fell into category 3 (Table 2) (Yang et al., 2013). The number of target genes found in the *phyB* mutant library was similar to that identified in the WT library, but their reliability was higher. Four targets cleaved by four novel rice-specific miRNAs in the *phyB* library belonged to categories 0 and 2, respectively (Table 2 and Additional file 6). In total, 24 genes (30 transcripts) were found to be sliced by 24 novel rice-specific miRNAs in the two libraries (Table 2). The annotations of these target genes indicated that they might be involved in the regulation of multiple biological processes, such as transcription, transport, carbohydrate metabolism, and protein degradation.

**Table 2:** **Targets of novel rice-specific miRNAs identified by degradome sequencing**.

**miR_name**	**Target (transcript)**	**Annotation**	**Alignment score**	**Cleavage site**	**Reads at cleavage site (tpb) (WT)**	**Reads at cleavage site (tpb) (*phyB*)**	**Category (WT)**	**Category (*phyB*)**
PC-5p-35252_76	LOC_Os01g34200.2	Apoptosis antagonizing transcription factor	4	806	0	31.78		4
PC-5p-35252_76	LOC_Os01g34200.1	Apoptosis antagonizing transcription factor	4	814	0	31.78		4
PC-5p-3668_482	LOC_Os07g30690.1	indole-3-acetate beta-glucosyltransferase	2.5	1926	56.48	0	4	
PC-5p-3668_482	LOC_Os03g08530.1	Alanine aminotransferase 2	3	2066	56.48	0	4	
PC-5p-424321_3	LOC_Os06g38210.2	Expressed protein	4	609	112.96	0	4	
PC-5p-444516_3	LOC_Os06g50146.1	Calcium-dependent protein kinase CPK1 adapter protein 2	4	1391	112.96	0	4	
PC-5p-452526_3	LOC_Os07g49480.2	Kinase interacting protein 1	1.5	4243	112.96	0	4	
PC-5p-452526_3	LOC_Os03g12620.2	O-Glycosyl hydrolases family 17 protein	4	1764	56.48	0	4	
PC-5p-452526_3	LOC_Os03g12620.1	O-Glycosyl hydrolases family 17 protein	4	1776	56.48	0	4	
PC-5p-45977_56	LOC_Os03g12620.2	O-Glycosyl hydrolases family 17 protein	2	1750	0	63.56		2
PC-5p-45977_56	LOC_Os03g12620.1	O-Glycosyl hydrolases family 17 protein	2	1762	0	63.56		2
PC-5p-47661_56	LOC_Os09g39910.2	ABC transporter	4	2264	0	21.19		4
PC-5p-47661_56	LOC_Os09g39910.3	ABC transporter	4	2351	0	21.19		4
PC-5p-47661_56	LOC_Os09g39910.1	ABC transporter	4	2289	0	21.19		4
PC-5p-48672_55	LOC_Os11g24070.1	Lipid transfer protein 12	3.5	223	75.30	42.37	3	3
PC-5p-553057_2	LOC_Os09g12230.1	Ubiquitin-conjugating enzyme 28	3.5	1006	0	21.19		3
PC-5p-553057_2	LOC_Os02g43560.1	WRKY family transcription factor	3.5	2743	0	7.94		4
PC-5p-58861_41	LOC_Os09g32940.2	Expressed protein	2	1683	0	63.56		4
PC-5p-6540_314	LOC_Os01g73250.1	Abscisic stress-ripening	4	1127	255.91	63.56	3	4
PC-5p-68238_35	LOC_Os03g12620.2	O-Glycosyl hydrolases family 17 protein	0	1750	0	63.56		2
PC-5p-68238_35	LOC_Os03g12620.1	O-Glycosyl hydrolases family 17 protein	0	1762	0	63.56		2
PC-5p-69000_33	LOC_Os03g12620.2	O-Glycosyl hydrolases family 17 protein	2.5	1750	0	63.56		2
PC-5p-69000_33	LOC_Os03g12620.1	O-Glycosyl hydrolases family 17 protein	2.5	1762	0	63.56		2
PC-5p-69222_34	LOC_Os09g32940.2	expressed protein	4	1683	0	63.56		2
PC-5p-79829_27	LOC_Os09g30454.1	OsWAK87 - OsWAK receptor-like protein kinase	4	1193	45.18	25.42	3	3
PC-5p-804152_2	LOC_Os04g32340.1	RNA-binding motif protein	3	3732	0	127.11		2
PC-5p-818018_4	LOC_Os01g40420.2	Cystathionine beta-synthase (CBS) protein	4	644	0	127.11		0
PC-5p-838756_2	LOC_Os03g48010.1	Exostosin	4	2681	56.48	0	4	
PC-5p-84112_25	LOC_Os02g40870.2	Phosphatidylinositol N-acetylglucosaminyltransferase subunit C	2	1377	0	190.67		0
PC-5p-84112_25	LOC_Os02g40870.1	Phosphatidylinositol N-acetylglucosaminyltransferase subunit C	2	1437	0	190.67		0
PC-5p-84112_25	LOC_Os09g21689.2	Expressed protein	4	4321	0	31.78		4
PC-5p-84112_25	LOC_Os09g21689.3	Expressed protein	4	6110	0	31.78		4
PC-5p-8961_251	LOC_Os07g08669.1	Expressed protein	3.5	757	112.96	0	4	
PC-5p-90987_22	LOC_Os02g32930.1	Sodium Bile acid symporter family	4	1291	112.96	0	4	
PC-5p-96214_21	LOC_Os01g15110.1	Syntaxin/t-SNARE family protein	4	898	0	63.56		4
PC-5p-96928_24	LOC_Os02g05450.1	Pathogenesis related homeodomain protein A	3	1644	112.96	0	4	
PC-5p-99353_20	LOC_Os08g16255.1	CPSF6 protein	3	1709	112.96	0	4	

tpb, tags per billion.

### Targets of differentially expressed miRNAs between the WT and *phyB* libraries

In this study, 135 miRNAs were found to be differentially expressed between the WT and *phyB* libraries (Additional file 4). The identification and analysis of genes cleaved by the differentially expressed miRNAs could provide useful information to understand the roles of these differentially expressed miRNAs in phyB-mediated light signaling.

**Figure 5:**
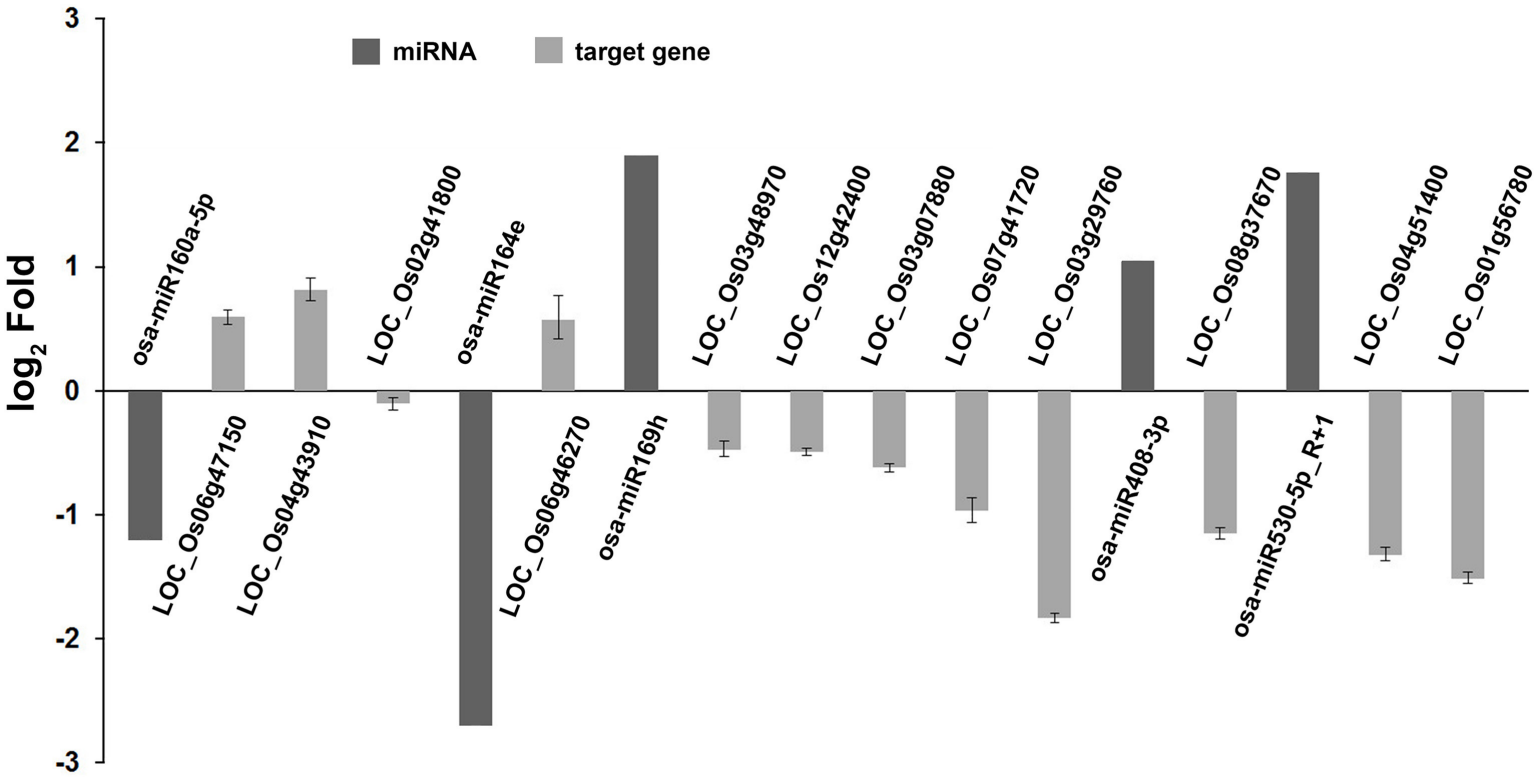
**Validation of degradome sequencing result by qRT-PCR**. The expression of five miRNAs and 12 their corresponding targets identified by degradome sequencing were shown in this histogram. The miRNA expression values were obtained from high-throughput sequencing. Expression values of their corresponding targets were obtained from qRT-PCR results. Total RNAs were isolated from the fourth leaves of WT and *phyB* mutant at the five-leaf stage. Expression of targets was determined by qRT-PCR. *OsEF-1α* was used as a normalization control. Bars indicate the SD across three PCR reactions. For each target gene or miRNA, the ratio of *phyB*/WT was log2 transformed to draw his histogram.

**Figure 6:**
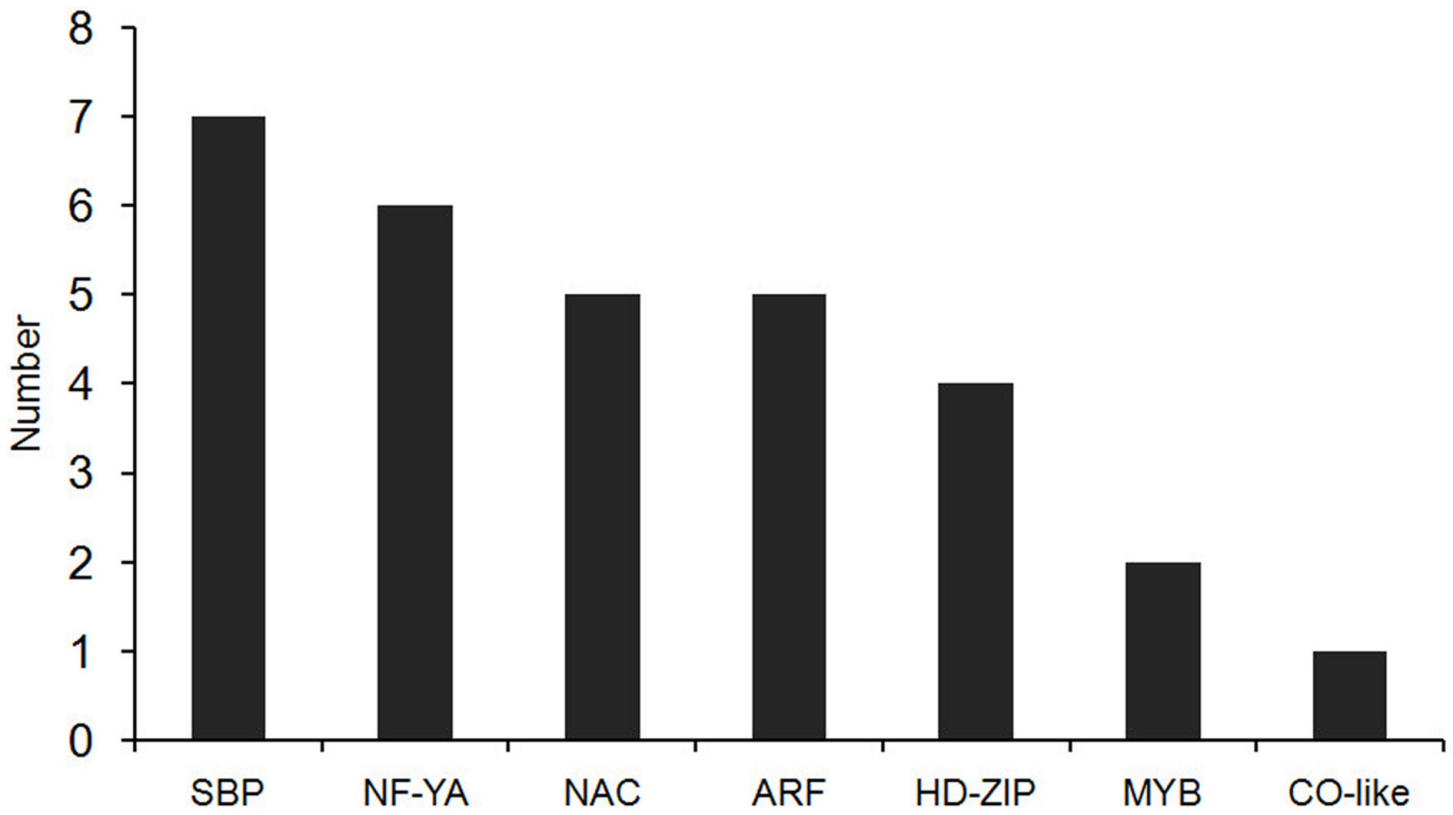
**The distribution of transcription factors targeted by differentially expressed miRNAs**. Thirty transcription factors targeted by differentially expressed miRNAs belong to seven types of transcription factor family.

In our degradome sequencing results, 32 of the differentially expressed miRNAs were found to target 70 genes (Additional file 8). To validate the results from degradome sequencing, the expression patterns of 12 genes targeted by five differentially expressed miRNAs were examined in the WT and *phyB* mutant by qRT-PCR. As shown in Figure 5, the expression patterns of most of these targets were opposite to that of the corresponding miRNAs, except for LOC_Os02g41800. GO analysis of genes targeted by differentially expressed miRNAs revealed that genes with transcription factor activities and related to regulation of transcription were over-represented (Additional file 9). Based on the annotations of all the targets cleaved by the differentially expressed miRNAs, we found that a large number of them (30/70) belonged to seven different transcription factor families that play important roles in plant growth and development, and stress responses (Figure 6, Additional file 8). For example, osa-miR160a-5p targeted four members of the ARF transcription factors, osa-miR169h targeted six NF-YA transcription factors, and osa-miR164d and osa-miR164e targeted five and four members of the NAC transcription factors, respectively (Additional file 8). Among them, 23 genes encoding transcription factors were sliced by 11 known differentially expressed miRNAs, and 11 genes encoding transcription factors were sliced by five novel differentially expressed miRNAs. Four HD-ZIP transcription factors were the common targets of known miRNA osa-miR166k-3p and novel miRNA sbi-miR166a_R+1_1ss1TG (a miRNA that has one base more than known sbi-miR166a in the 3′-end, and with one substitution of T->G at position 1). NF-YA, NAC, ARF, MYB, and CO-like transcription factors were targeted by known miRNAs. SBP transcription factors were only found to be targeted by novel miRNAs (ath-miR156g, bdi-miR156b-5p_R+1, sbi-miR156e_R+1_1ss14CT, bdi-miR156b-5p_L+1_1ss15TG) that show high sequence similarity to miR156 in *Arabidopsis*, *Brachypodium distachyon*, and *Sorghum bicolor* (Additional file 8), but have differences from the known members of miR156 family in rice.

In addition, a gene (LOC_Os01g56780) targeted by osa-miR530-5p_R+1 which is up-regulated significantly in the *phyB* mutant (Additional file 8) encodes a protein with high similarity to the tandem zinc knuckle/PLU3 domain protein TZP in *Arabidopsis* (Additional file 8). TZP is a downstream component of the circadian clock and light signaling and participates in regulating seedling development directly (Loudet et al., 2008). The identification of this TZP homologous gene indicated that osa-miR530-5p_R+1 might participate in phyB-mediated light signaling via regulating the expression of the zinc knuckle/PLU3 domain protein.

## Discussion

### miRNAs expressed in the WT and *phyB* mutant

Traditional methods, such as direct cloning and sequencing, 5′ RACE and northern blotting have been used to identify a number of miRNAs and their targets. However, it is difficult to identify novel miRNAs and their targets that are expressed at low levels through these methods, and the experimental procedures are complex and relatively time-consuming (Bonnet et al., 2004; Adai et al., 2005; Zhang et al., 2005; Unver et al., 2009). In contrast, next-generation sequencing combined with degradome analysis provides a rapid and high-throughput tool to identify novel, differentially expressed miRNAs and their targets. Therefore, it has been used widely in the identification of miRNAs and their targets in various plant species (Hao et al., 2012; Mao et al., 2012; Karlova et al., 2013; Yang et al., 2013). Although miRNAs play central roles in plant growth and development, and environmental stresses responses (Jones-Rhoades et al., 2006; Mallory and Vaucheret, 2006; Shukla et al., 2008; Voinnet, 2009; Sunkar et al., 2012), few studies have been carried out to identify miRNAs involved in phyB-mediated light signaling (Martin et al., 2009). The present study is the first report to identify miRNAs that participate in phyB-mediated light signaling in rice at genome level.

In total, 720 known and 704 novel miRNAs were identified from WT rice and the *phyB* mutant. Most of known miRNAs fell into two categories, 21 nt miRNA (31.8%) and 24 nt miRNA (39.2%) (Figure 1C), and the latter one constituted the largest category (72.4%) of novel miRNAs (Figure 1C). The evolutionary histories of these two kinds of miRNAs are different. Previous study revealed that the 21 nt miRNAs were derived from the ancient *MIR* genes that cleaved by Dicer-like 1 (DCL1), and the 24 nt miRNAs were mainly generated from recently evolved *MIR* genes cleaved by DCL3 (Vazquez et al., 2008). The functions of 24 nt miRNAs are poorly-studied relative to those of canonical 21 nt miRNAs, but they were observed to be regulated developmentally and some of them were conserved during evolution (Vazquez et al., 2008), indicating that they probably have biological functions as that of 21 nt miRNAs. Additionally, most known miRNAs identified in this study were p5/p3 strands of documented miRNAs (469, 65.1%), which usually have lower stability than that of other miRNAs in cells and expressed at relative low levels (Jones-Rhoades et al., 2006; Rajagopalan et al., 2006). Our results were in accordance with this pattern, 60.3% (283) of p3/p5 strands of known rice miRNAs were expressed at low levels (the normalized expression value ≤10) in both the WT and *phyB* mutant (Additional file 2a). However, 34 p3/p5 strands of known rice miRNAs were expressed at relative high levels (the normalized expression value ≥100) in at least one library (Additional file 2a), especially osa-MIR397b-p5, osa-MIR5794-p3, and osa-MIR7695-p3, which indicate they may be true mature miRNAs.

Comparative analysis of miRNAs expressed between the WT and *phyB* mutant identified 135 differentially expressed miRNAs, including 97 up-regulated and 38 down-regulated miRNAs, which are candidate miRNAs for phyB-mediated light signaling (Additional file 4). Few studies focus on the miRNAs roles in phyB-mediated light signaling previously except for one study that indicates a bridge between miRNA and the phyB-mediated light signaling pathway (Martin et al., 2009). PHYB acts as a repressor of tuberization under long days in potato, and silencing of *PHYB* completely abolishes this repression (Jackson et al., 1996). A light-regulated miRNA, miR172, is also involved in tuberization regulation in a photoperiod-dependent manner in potato (Martin et al., 2009). In addition, the expression of miR172 seems to be under the control of *PHYB*. In anti-*PHYB* potato, the expression of miR172 is reduced in leaves but increased in stolons, suggesting miR172 acts downstream of phyB to regulate tuberization in response to day length (Martin et al., 2009). In this study, four members of osa-miR172 family (osa-miR172a/b/c/d) were also detected in both the WT and *phyB* libraries (Additional file 2a), and nine genes were identified as the targets of osa-miR172 family (Additional file 6). However, the osa-miR172 family members showed no significantly differential expression (Additional file 2a). This may be caused by the functional divergence of miRNAs between monocots and dicots.

Further molecular and genetic studies focusing on the functions of these miRNA should provide new clues to build bridges between miRNA and the phyB-mediated light signaling pathway.

### Transcription factors, the main regulator of plant life activities, are probably involved in phyB-mediated light signaling under the control of miRNAs

Compared to traditional target finder tools, degradome sequencing has unmatchable advantages. Thousands of genes targeted by miRNAs could be detected in one sequencing reaction, even if their expression levels are low. Analysis of degradome sequencing results of the WT and *phyB* mutant showed that at least 1957 genes (2893 transcripts) were targeted by miRNAs in rice (Additional file 6). The identification of the large number of targets in this study will provide a basis and new clues for illuminating miRNA roles in rice growth and development.

In this study, 70 genes were found to be targeted by 32 of the differentially expressed miRNAs (Additional file 8). Among them, 30 out of 70 target genes are encoding transcription factors. GO analysis revealed that transcription factors were significantly enriched in the genes targeted by differentially expressed miRNAs (Additional file 9). These results are in agreement with the previous finding (Jones-Rhoades et al., 2006). Seven types of transcription factors were identified as targets of differentially expressed miRNAs between WT and *phyB* mutant, including members of CO-Like, MYB, HD-ZIP, ARF, NAC, NF-YA, and SBP families (Figure 6, Additional file 8). In *Arabidopsis*, the members of several transcription factor families mentioned above have been reported to be involved in phytochrome-mediated light signaling. CO (Constans) protein is a transcription factor containing two B-box zinc fingers at the N-terminus and a CCT domain in C-terminus, and its stability is regulated by phytochrome-mediated light signaling. PhyA stabilizes the CO protein and promotes flowering, whereas phyB promotes CO degradation and delays flowering (Yanovsky and Kay, 2002; Cerdan and Chory, 2003; Valverde et al., 2004). ATHB-2 (*Arabidopsis thaliana* homobox protein 2), an HD-ZIP type transcription factor, was also found to be involved in phytochrome-mediated light signaling. The expression of ATHB-2 is regulated by changes in the ratio of red to far-red light, and it acts as a negative regulator of type II phytochromes (Carabelli et al., 1996; Steindler et al., 1999). The phenotypes of transgenic *Arabidopsis* with over-expressed *ATHB-2* are the same to those of *phyB* null mutant. Consistently, *ATHB-2* is expressed at relative high levels in all tissues and organs both in the day and night in *phyB* mutant (Carabelli et al., 1996; Steindler et al., 1999). A MYB family transcription factor, CCA1 (circadian clock associated 1) plays an important role in the circadian clock and phytochrome-regulated morphogenesis in *Arabidopsis* (Wang et al., 1997; Wang and Tobin, 1998). In addition, LAF1 (long after far-red light 1), HFR1 (long hypocotyl in far-red 1), and HY5 (elongated hypocotyl 5), which are members of MYB, basic helix-loop-helix (bHLH) and basic leucine zipper (bZIP) transcription factor families, respectively, act independently to transmit phyA signals downstream (Jang et al., 2013). In this context, we deduced that transcription factors targeted by the differentially expressed miRNAs in this study are probably involved in phyB-mediated light signaling in rice.

## Concluding remarks

In this study, high-throughput small RNA sequencing and degradome sequencing were performed on libraries constructed from the WT and *phyB* mutant leaves in rice. As a result, 135 miRNAs exhibited differential expression between the WT and *phyB*. Among them, 32 differentially expressed miRNAs were found to slice 70 genes. A large proportion of target genes (30/70) encode members of transcription factor families. Although some members of these transcription factor families have been reported to be involved in phytochrome-mediated signaling pathway in *Arabidopsis*, we will dissect whether and how these transcription factors identified in this study function in phyB-mediated photomorphogenesis and stress responses in rice in the future. Nonetheless, our findings, for the first time, provide a genome-wide link between microRNAs and phyB-mediated light signaling pathway.

## Author contributions

XX conceived and designed the study. WS, XW, YW, XL, and HS performed molecular experiments (material collection, RNA extraction, real-time PCR). WS and XHX carried out data analysis. XX and XHX drafted this manuscript. XX and XW revised the manuscript. All the authors read and approved the final manuscript.

### Conflict of interest statement

The authors declare that the research was conducted in the absence of any commercial or financial relationships that could be construed as a potential conflict of interest.

## Supplementary material

Additional file 1 **Primers used for qRT-PCR**.

Additional file 2 **Known and novel miRNAs identified from the WT and *phyB* libraries**. 2a. known miRNAs identified by high-throughput sequencing in WT and *phyB* mutant. 2b, novel miRNAs identified by high-throughput sequencing in WT and *phyB* mutant.

Additional file 3 **Analysis of the first nucleotides of known and novel miRNAs identified in this study**.

Additional file 4 **Differentially expressed miRNAs between the WT and *phyB***.

Additional file 5 **Distribution of reads from degradome sequencing**.

Additional file 6 **Targets identified from degradome sequencing**.

Additional file 7 **GO analysis of targets obtained from degradome sequencing**.

Additional file 8 **Targets of differentially expressed miRNAs between the WT and *phyB***.

Additional file 9 **GO analysis of genes targeted by differentially expressed miRNAs**. Boxes in the graph represent GO terms labeled by their GO ID, term definition and statistical information. The significant term (adjusted P ≤ 0.05) are marked with color, while non-significant terms are shown as white boxes. The degree of color saturation of a box is positively correlated to the enrichment level of the term. Solid, dashed, and dotted lines represent two, one and zero enriched terms at both ends connected by the line, respectively. The rank direction of the graph is set to from top to bottom.
