# Supplementary figures and images for: Genome-wide identification of microRNAs and their targets in wild type and phyB mutant provides a key link between microRNAs and the phyB-mediated light signaling pathway in rice
Source: Front Plant Sci. 2015 May 29;6:372. doi: 10.3389/fpls.2015.00372 (PMC4448008; doi:10.3389/fpls.2015.00372)

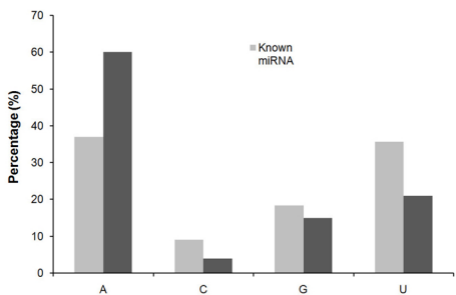

Supplement: Additional file 3 — Analysis of the first nucleotides of known and novel miRNAs identified in this study. [file DataSheet3.PDF]

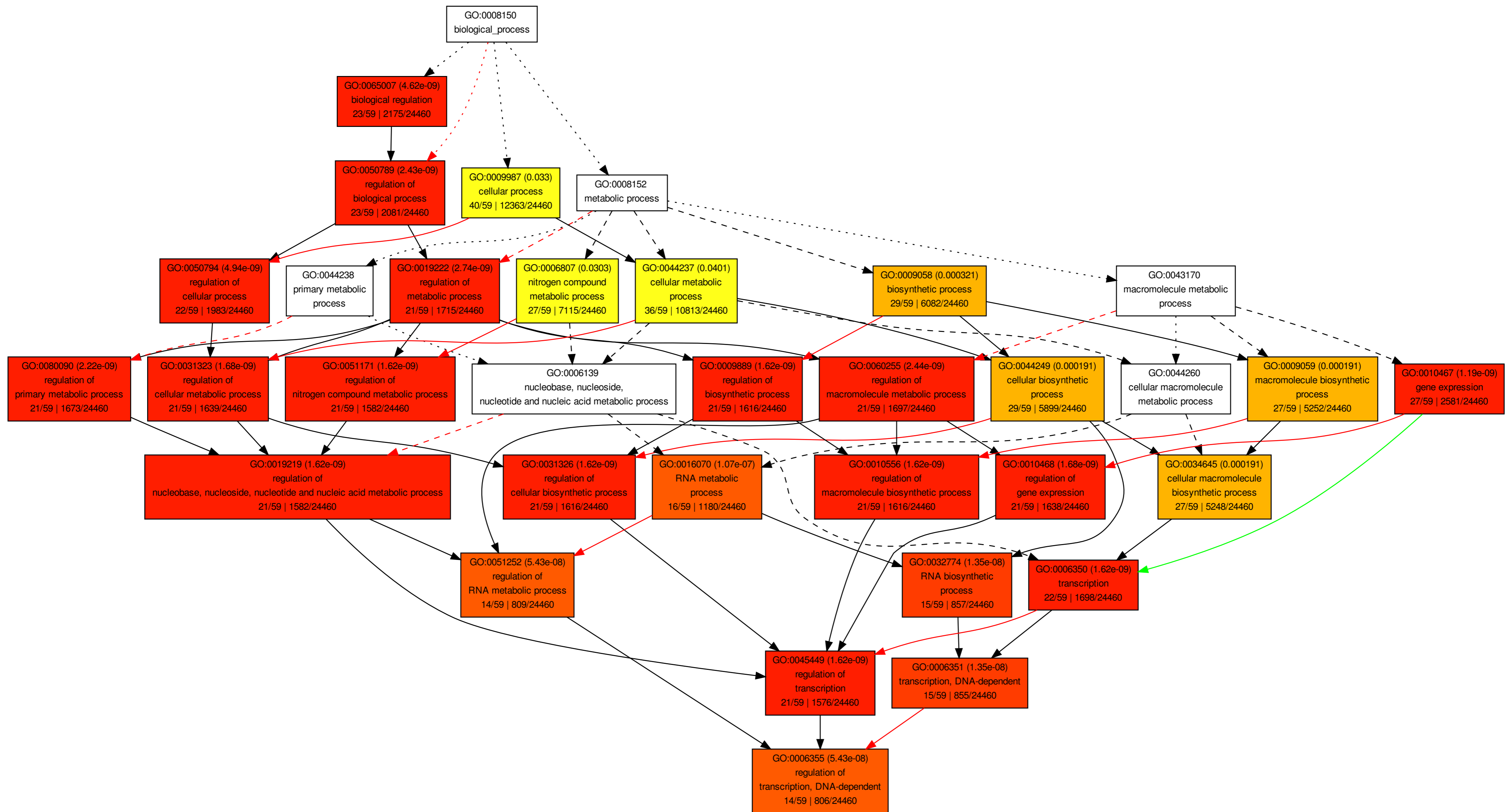

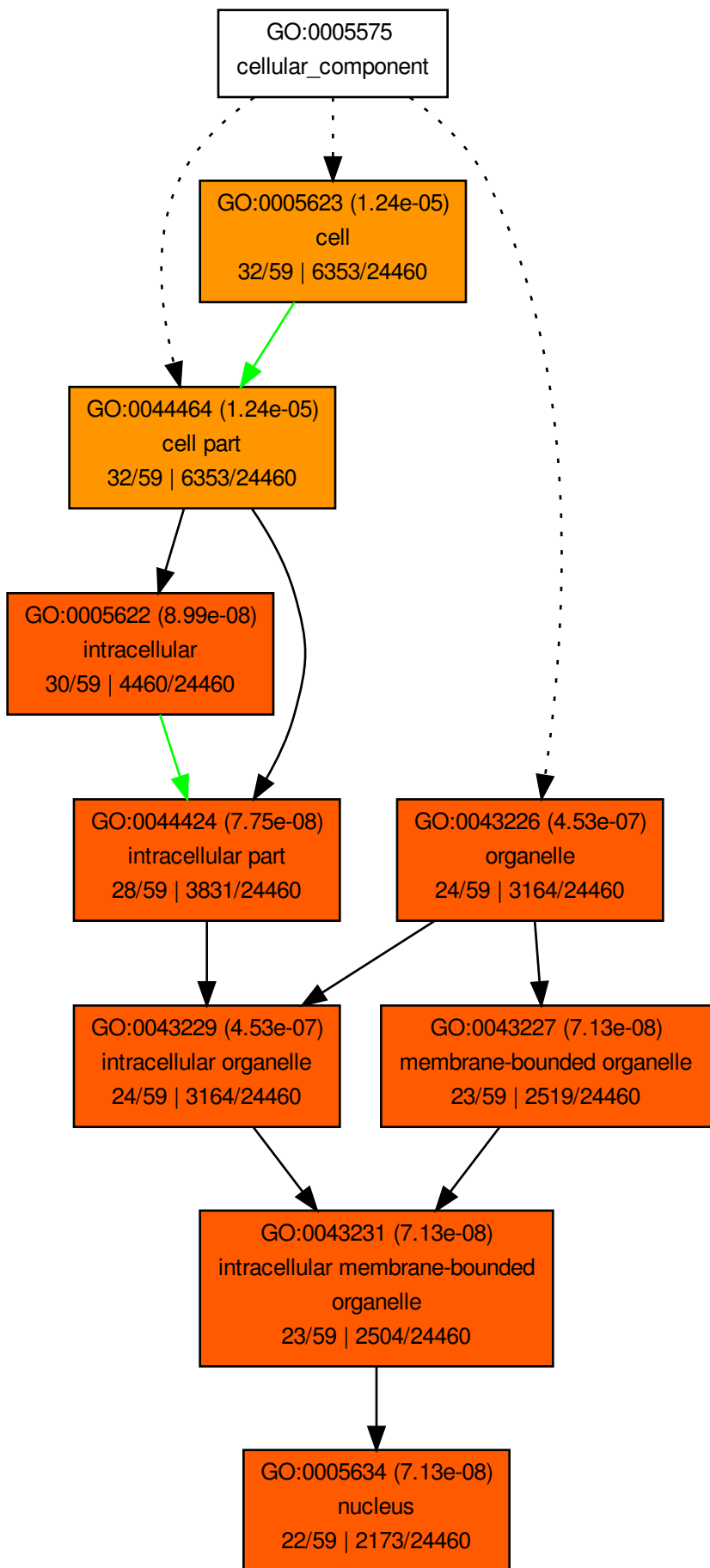

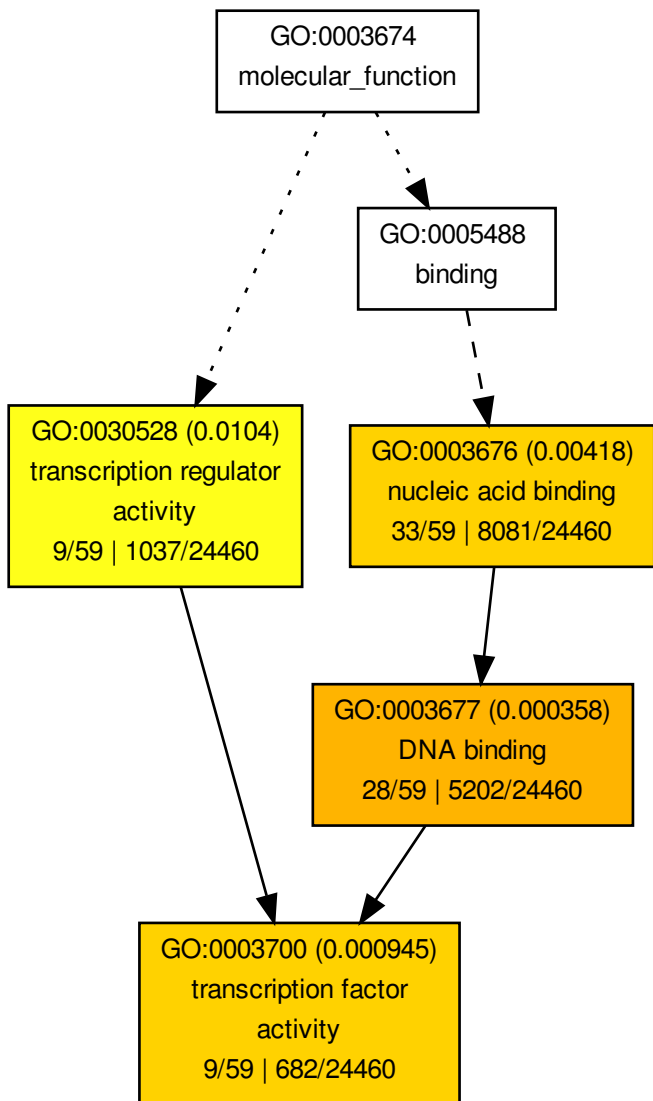

Supplement: Additional file 9 — GO analysis of genes targeted by differentially expressed miRNAs. Boxes in the graph represent GO terms labeled by their GO ID, term definition and statistical information. The significant term (adjusted P ≤ 0.05) are marked with color, while non-significant terms are shown as white boxes. The degree of color saturation of a box is positively correlated to the enrichment level of the term. Solid, dashed, and dotted lines represent two, one and zero enriched terms at both ends connected by the line, respectively. The rank direction of the graph is set to from top to bottom. [file DataSheet9.PDF]
